# Identification of Novel Gene Signature Associated with Cell Glycolysis to Predict Survival in Hepatocellular Carcinoma Patients

**DOI:** 10.1155/2021/5564525

**Published:** 2021-05-06

**Authors:** Ming Wang, Feng Jiang, Ke Wei, Erli Mao, Guoyong Yin, Chuyan Wu

**Affiliations:** ^1^Department of Plastic and Burn Surgery, The First Affiliated Hospital of Nanjing Medical University, Nanjing 210000, China; ^2^Pediatric Department, The First Affiliated Hospital of Nanjing Medical University, Nanjing 210000, China; ^3^Neonatal Department, Obstetrics and Gynecology Hospital of Fudan University, Shanghai 200011, China; ^4^Medical Service Section, The First Affiliated Hospital of Nanjing Medical University, Nanjing 210000, China; ^5^Department of Rehabilitation Medicine, The First Affiliated Hospital of Nanjing Medical University, Nanjing 210000, China; ^6^Department of Orthopedics, The First Affiliated Hospital of Nanjing Medical University, Nanjing 210000, China

## Abstract

**Purpose:**

As hepatocellular carcinoma (HCC) is a complex disease, it is hard to classify HCC with a specific biomarker. This study used data from TCGA to create a genetic signature for predicting the prognosis of HCC patients.

**Methods:**

In a group of HCC patients (*n* = 424) from TCGA, mRNA profiling was carried out. To recognize gene sets that differed significantly between HCC and normal tissues, an enrichment study of genes was carried out. Cox relative hazard regression models have been used to identify genes that are significantly associated with overall survival. To test the function of a prognostic risk parameter, the following multivariate Cox regression analysis was used. The log-rank test and Kaplan–Meier survival estimates were used to test the significance of risk parameters for predictive prognoses.

**Results:**

Eight genes have been identified as having a significant link to overall survival (PAM, NUP155, GOT2, KDELR3, PKM, NSDHL, ENO1, and SRD5A3). The 377 HCC patients were divided into eight-gene signature-based high/low-risk subgroups. The eight-gene signature's prognostic ability was unaffected by a number of factors.

**Conclusion:**

To predict the survival of patients with HCC, an eight-gene signature associated with cellular glycolysis was then identified. The findings shed light on cellular glycolysis processes and the diagnosis of patients with low HCC prognoses.

## 1. Introduction

Hepatocellular carcinoma (HCC) is the most prevalent primary type of liver cancer [1, 2]. This is a heterogeneous tumor with multiple genetic and epigenetic events and is typically associated with particular risk factors such as hepatitis B or C infection, excessive consumption of alcohol, hemochromatosis, or nonalcoholic fatty liver disease induced by insulin resistance and obesity [3]. It is also the world's fifth most prevalent cancer and the second most influential cause of cancer mortality in people. Liver cancer accounts for 70–85 percent of the overall economic burden of cancer [4, 5]. While some improvement in its clinical diagnosis and treatment has been made in recent years, HCC's metastasis and recurrence rates after radical resection are still high. Specific diagnosis criteria and clinical targets are also desperately required for this disorder.

In the modern age of “omics,” the advent of a number of innovative techniques such as sequencing and microarray also accelerated the quest for biomarkers [6–8]. Many biomarkers of HCC have been identified, such as *α*-fetoprotein (AFP) and des-*γ*-carboxy prothrombin (DCP) [9]. Via database mining, we established thousands of biomarkers which may be correlated with tumor patient prognosis. Since HCC is a dynamic disorder, HCC with a specific biomarker is also challenging to characterise. Studies have shown that analyzing genetic traits affecting several genes may boost prediction of prognoses [10, 11]. Specific treatment strategies can be guided by the polygenic prognostic characteristics of primary tumor biopsy. Latest research also investigated the impact of polygenic markers on HCC to determine prognosis and classify prospective patients with high-risk HCCs.

Aerobic glycolysis is one of the important characteristics of tumor, which provides survival advantage for tumor. At present, most people think that malignant tumor is not only a genetic disease but also an energy metabolic disease [10, 12]. Many glycolytic enzymes can stimulate cancer cell growth, and this “Warburg effect” is reported in various tumor forms [13, 14]. The creation of a new gene signature correlated with glycolysis may also forecast HCC. Genes were selected in this study using the gene set enrichment analysis (GSEA). GSEA is a new computing method, which can reveal more general trend of data, rather than just detect gene expression differences [15]. This approach thus strengthens the mathematical study of biological speech and biological context.

In our analysis, we collected glycolysis-related genomes from 424 HCC cases with full TCGA database mRNA expression datasets. We have verified the primary glycolysis-related mRNAs and built up an eight-gene risk signature that can predict patient prognosis accurately. Interestingly, this signature of the risk associated with glycolysis will accurately identify patients in the high-risk community who have low prognosis on multiple pathways.

## 2. Methods

### 2.1. Patients' Clinical and mRNA Expression Data Collection

In TCGA (https:/portal.gdc.cancer.gov/), we have collected clinical evidence and mRNA expression profiles from the hepatocellular cancer patients [16]. The trial included clinical data from 377 patients and age, gender, grade, stage, topography of the tumor (*T*), distant metastasis (*M*), and lymph node status (*N*) (Table 1).

### 2.2. Gene Set Enrichment Analysis

We selected five gene sets that are most closely related to glycolysis for GSEA (http://www.broadinstitute.org/gsea/index.jsp) analysis to determine whether there are significant differences in the recognized gene sets between the HCC group and normal group [17]. First, the expression level of 56753 mRNAs in the liver and neighboring tissues was examined. Finally, we determined the function of the follow-up analysis with the standardized *P* value (*P* < 0.05).

### 2.3. Data Processing and Calculation of Risk Parameters

The log2 transformation was used to normalize single mRNA from the expression profiles. Univariate Cox regression analysis has been used to identify genes correlated with total survival (OS) and has been exposed to multivariate Cox regression to test prognostic genes and gain coefficients. Selected mRNAs have then been divided into the type and protective type (0 < HR < 1) (hazard ratio, HR> 1). Risk parameter = ∑ (*βn* × expression of gene *n*). The 548 patients were grouped into high-risk and low-risk subgroups utilizing the median risk criterion as a cutoff.

### 2.4. Statistical Analysis

Kaplan–Meier survival curves have been used to measure the value of the risk parameter. Multivariate Cox analysis and stratification data analysis were performed to check whether the risk parameters were independent of clinical characteristics such as age, gender, grade, stage, tumor topography (*T*), distant metastasis status (*M*), and lymph node status (*N*). *P* < 0.05 was found statistically important. *R* software (v3.6.1) was used for all statistical analysis.

## 3. Results

### 3.1. Initial Gene Screening Using GSEA

**Figure 1:**
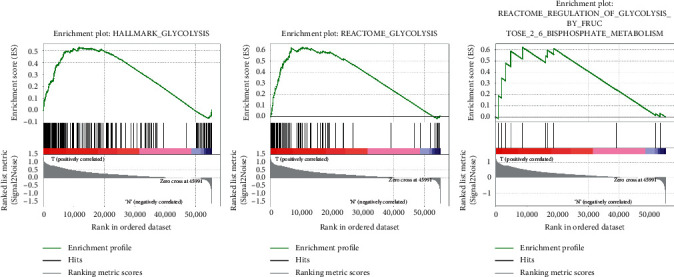
Enrichment plots of 3 glycolysis gene sets which had a significant difference between noncancerous tissues and HCC tissues by performing GSEA.

**Figure 2:**
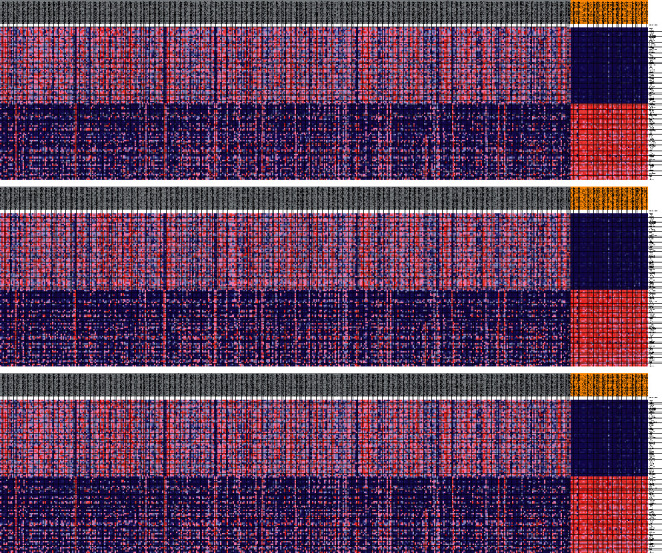
Selected gene sets in five genes.

From the TCGA report, we received clinical features from 377 HCC patients along with expression details for 56753 mRNAs. The expressive signatures of the glycolysis gene sets have been derived from the MSigDB database by concentrating multiple gene sets. GSEA was performed to decide if the gene sets detected differed considerably between tumor tissues and normal tissues. 3 gene sets, including the Hallmark, Reactome, and Reactome modulation of glycolysis, were significantly enriched with standardized *P* values <5% of the five gene sets most correlated with glycolysis (Table 2 and Figures 1 and 2).

### 3.2. Identification of Survival-Associating mRNAs Related to Glycolysis

First, the univariate Cox regression study was carried out with 226 genes for early screening, and 201 genes (*P* < 0.05) were collected. A multivariate Cox regression study was subsequently performed to further explore the relation between the expression profiles of 201 mRNA and the survival of the individual, using the phased exclusion approach to classify the most relevant mRNAs. 31 mRNAs were verified, and eight of the 31 genes validated as independent prognostic markers of HCC are given in Table 3. The filtered mRNAs were classified into dangerous forms (PAM, NUP155, KDELR3, NSDHL, ENO1, and SRD5A3), with HR >1 associated with weaker survival and safe sort (GOT2 and PKM) and HR <1 associated with enhanced survival (Table 3).

**Figure 3:**
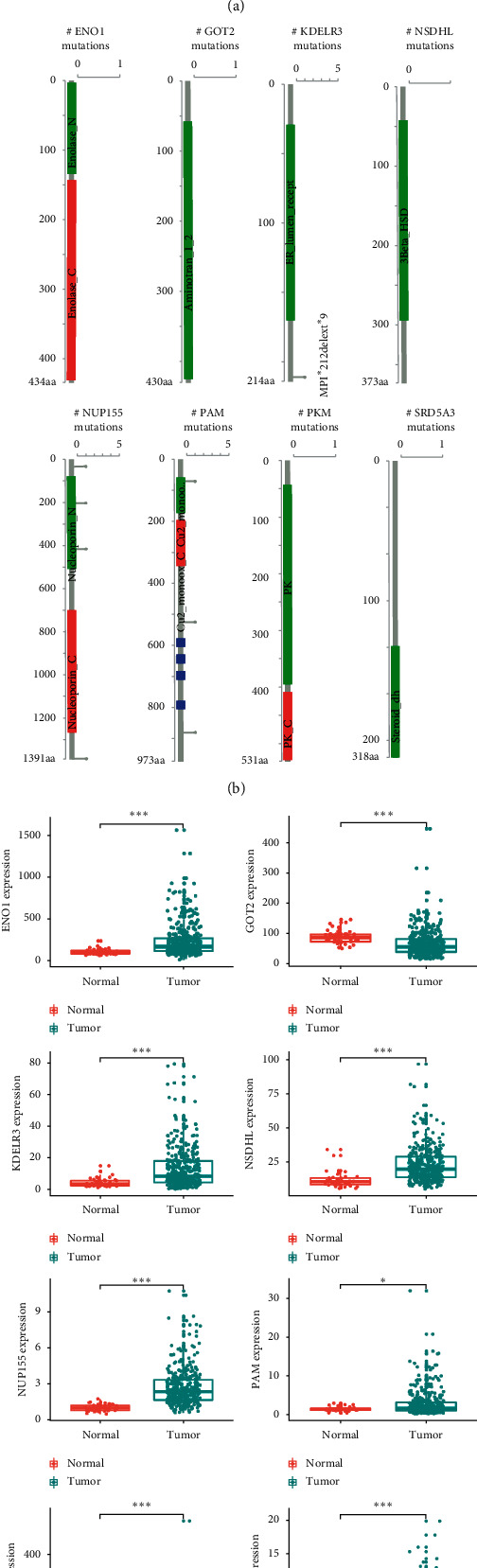
Identification of mRNAs related to patients' survival. (a) Selected genes' alteration in 377 clinical samples. (b) Selected genes' specific alteration in different pathological types of HCC. (c) Different expression of 8 selected genes.

The changes in eight filtered genes were then evaluated by analyzing 377 HCC samples in the database of cBioPortal (http:/cbioportal.org) [13]. The findings revealed that 35 (9.3%) of sequenced instances had changed the queried genes. The PAM gene included 3 missense mutations samples. The NUP155 gene included 3 missense mutations samples and 1 splice mutation sample. The KDELR3 gene was altered in 1.1% of cases. The PKM and SRD5A3 genes were modified in 0.3% of cases, with the NSDHL and ENO1 genes changed in 1.4% and 2.8% of cases, respectively (Figure 3(a)).

The expression differences between adjacent normal tissues and HCC tissues were also compared with 8 genes. The expression rates of the 8 genes have been greatly enhanced or reduced in HCC tissues (Figures 3(b) and 3(c)).

### 3.3. Construction of an 8-mRNA Signature to Predict Patient Outcomes

**Figure 4:**
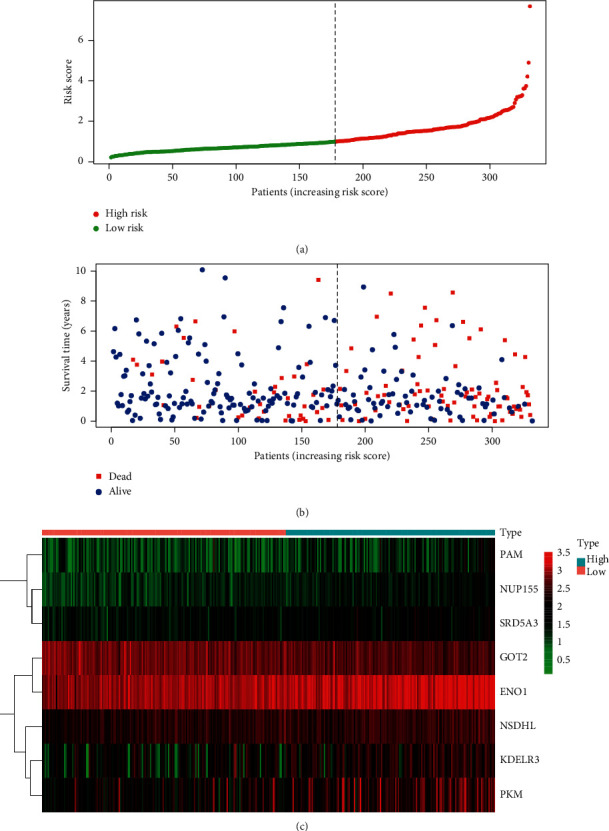
The eight-mRNA signature associated with risk parameter predicts OS in patients with endometrial cancer. (a) mRNA risk parameter distribution in each patient. (b) Survival days of EC patients in ascending order of risk parameters. (c) A heatmap of nine genes' expression profile.

**Figure 5:**
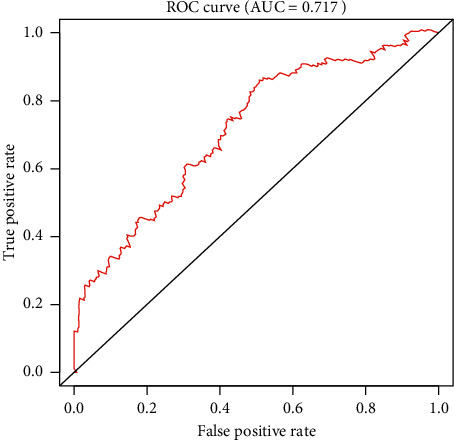
Receiver operating characteristic (ROC) analysis of the sensitivity and specificity of the risk score model.

The forecast score model was developed on the basis of a linear combination of weighted expression rates and regression coefficients from the Cox regression multivariate analysis: risk score = 0.2193 × expression of PAM + 0.4542 × expression of NUP155-0.2835 × expression of GOT2 + 0.1396 × expression of KDELR3-0.1785 × expression of PKM + 0.3203 × expression of NSDHL + 0.1829 × expression of ENO1 + 0.3133 × expression of SRD5A3. We estimated the outcomes and graded the patients by a mean risk value into high and low categories (Figure 4(a)). The life period of every patient (in years) is shown in Figure 4(b), and the high-risk patients showed higher mortality rates than the low-risk patients. In addition, a heatmap (Figure 4(c)) was released to show the expression profiles of the 8 mRNAs, utilizing the median risk score as a cutoff for patients to use the 8-mRNA survival risk score in a low-risk or high-risk category. The ROC curve review value was 0.717 (Figure 5), which showed that the 8-mRNA signature was well adapted and unique to the metastasis and survival of HCC patients. The amount of expression of dangerous mRNA (PAM, NUP155, KDELR3, NSDHL, ENO1, and SRD5A3) in the high-risk community was higher than that of the low-risk category. In comparison, in the high-risk community, the expression level mRNA type (GOT2 and PKM) was lower than in the low-risk category.

### 3.4. Risk Parameter Derived from 8-mRNA Signature is an Independent Prognostic Indicator

**Figure 6:**
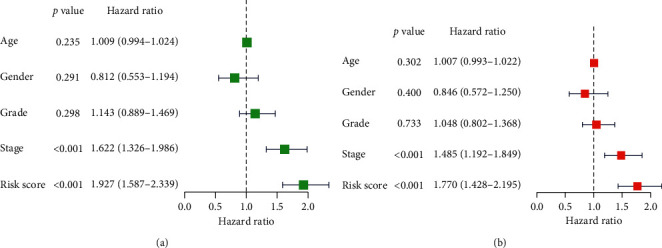
Univariable and multivariable analyses for each clinical feature. (a) Univariable analysis. (b) Multivariable analysis.

We have contrasted the prognostic meaning of risk parameters with clinical pathological parameters through univariable and multivariate analyses (Table 1). Samples were choosed with well-established clinical evidence. For the 377 HCC cases, the mean age was 65. Among the 377 patients, 255 (67.6%) were male and 122 (32.4%) were female. Among 372 patients, 235 (63.2%) had grade I-II tumors and 137 (36.8%) had grade III-IV tumors. Moreover, 262 (74.2%) of the 353 HCC patients suffered from stage I-II disease and 91 (25.8%) of the remaining patients suffered from stage III-IV disease. From the above, the risk parameter and stage were determined as independent prognostic indicators because these factors demonstrated significant variations both in univariate and in multivariate analyses (Table 4). The risk parameters displayed important *P* < 0.05 (HR = 1.770) prognostic values, in particular (Figure 6).

### 3.5. Validation of Eight mRNA Markers for Survival Prediction by Kaplan–Meier Curve Analysis

**Figure 7:**
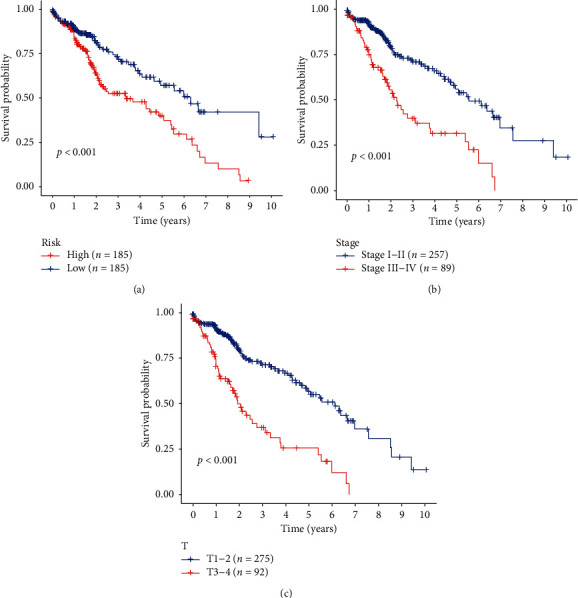
Kaplan–Meier survival analysis for HCC patients in TCGA dataset. (a) K–M survival curve for HCC patients with high/low risk. (b), (c) Clinical features including stage and tumor topography predict patients' survival.

Kaplan–Meier assessments of survival found that high-risk patients have a weak prognosis (Figure 7(a)). Univariate OS regression analysis of Cox found many predictive HCC-related clinicopathologic parameters, including age, gender, grade, stage, tumor topography, distant metastasis status, and lymph node status. We then used survival figures from Kaplan–Meier to test these findings, which provided clear outcomes, with weak prognosis correlated with patients that suffered from stage III-IV cancer and with tumor topography 3-4 (Figures 7(b) and 7(c)). These results further confirmed the reliability of the analysis.

**Figure 8:**
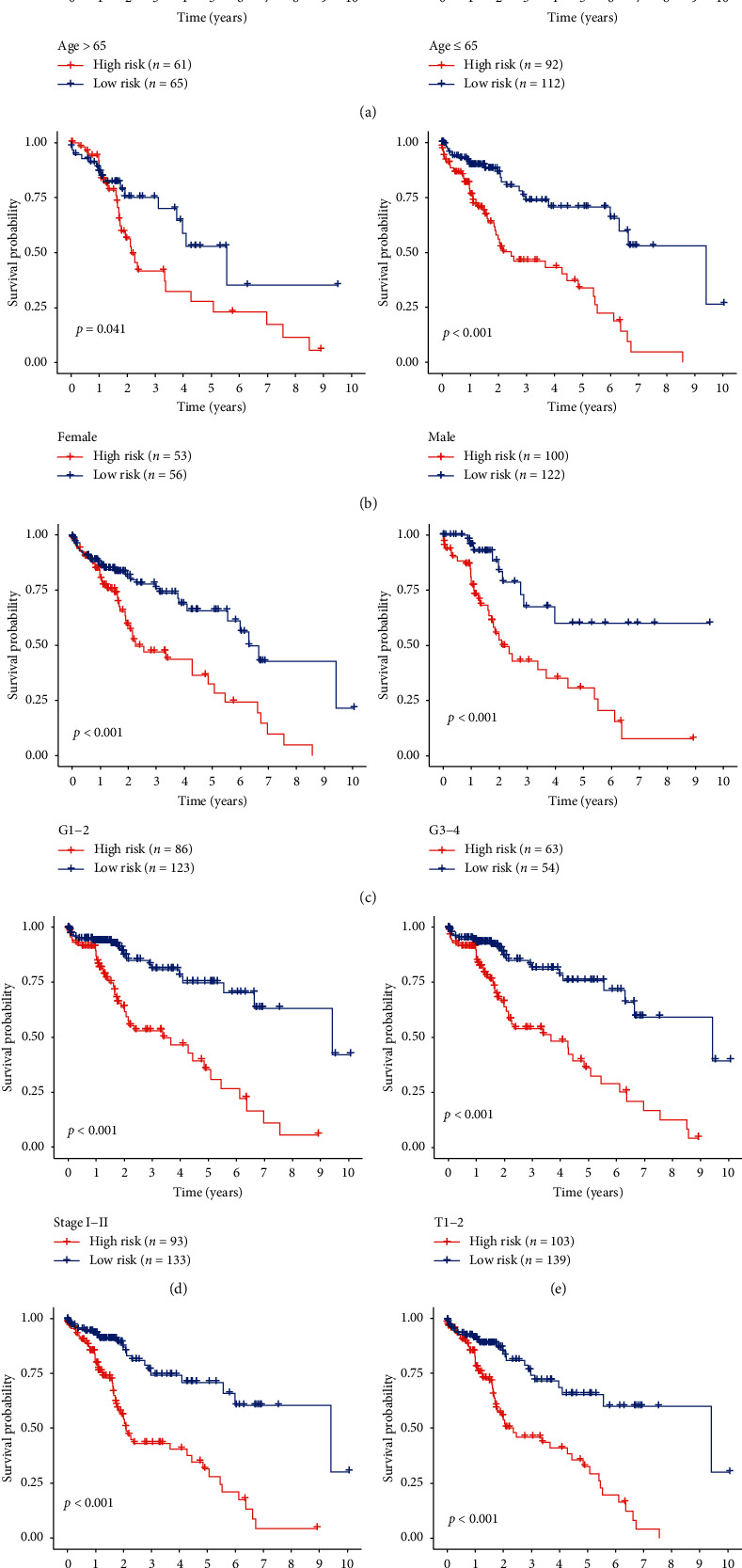
Kaplan–Meier curves for the prognostic value of risk parameter signature for the patients divided by each clinical feature. (a) Age, (b) gender, (c) grade, (d) stage I-II, (e) T1-2, (f) M0, and (g) N0.

Further stratified analysis for data processing has also been performed. As shown in the K–M curve, irrespective of age, class, or grade (e.g., grade I-II or grade III-IV); the eight-mRNA signature was a reliable prognostic marker for high-risk HCC patients with poor prognosis (Figure 8). The danger parameter cannot, however, be used separately for such subgroups in view of the specific subgroups of stage III-IV, T3-4. Maybe, it is because there are relatively few normal samples in these groups. That point calls for further exploration.

## 4. Discussion

In recent years, studies have shown that it is not accurate to use one or several clinical features to evaluate the prognosis of tumors. Therefore, more and more research studies focus on mRNA and regard it as a biological marker of tumor progression and prognosis. For example, AFP-L3 is considered to be a specific biomarker of HCC [18]. In the early stage of hepatocarcinogenesis, the expression of squamous cell carcinoma antigen (SCCA) complexed with IgM increased, which may be an important serum biomarker for early detection of hepatocarcinoma [19, 20]. Because gene expression is easily influenced by many factors, it cannot be used as a reliable and independent prognostic indicator in many cases. In fact, HCC is a complex disease, so it is difficult to use a single biomarker to determine its nature. Therefore, the study of binding biomarkers may provide valuable reference for diagnosis and prognosis. To boost the prediction, a mathematical model consisting of genetic markers of several linked genes along with the predictive influence of each constituent gene is used. This model is more reliable to determine the prognosis of tumor patients than a biomarker, so it is commonly used [21, 22].

The existing high-throughput sequencing technology can extract a large number of genomic data from a single sample to determine new diagnosis, prognosis, or pharmacological biomarkers [23]. And mathematical simulations have estimated the prognosis of certain cancers with the advancement of gene marker technologies. In patients with lung adenocarcinoma, for example, a new signature to inhibit metastasis and survival was discovered by means of Cox regression and ROC study. [10]. In this study, we identified three functional glycoside gene sets that are closely related to GSEA. As above, we have selected the top-level screening feature for glucose metabolism and survival in cancer patients. The prognostic value of 8 gene combinations was determined by univariate and multivariate Cox regression analyses. Compared with other known prognostic indicators, the selected risk model may be a more targeted and powerful prognostic evaluation method, which can be used as a more effective classification tool for patients with HCC.

We used TCGA's HCC data collection to gather genes linked to glycolysis and compare standard and HCC tissue results. Then, we selected Kaplan–Meier survival assessment, and the results showed that the prognosis of patients with low-risk parameters was relatively good. Among the eight genes, the expression of nup155 in HCC is considered to be part of the p53 regulatory network [24]. GOT2 has been shown to be involved in the energy metabolism of tumor cells. KDELR3 is considered to be one of genes that formed 11-gene-based prognostic signature of uveal melanoma [25]. PKM can promote anabolism and regulate glycolysis and promote tumorigenesis by glycolysis and control gene expression [26]. NSDHL is believed to be closely related to cholesterol metabolism. And in the study of glioma and lung cancer, ENO1 has been considered to be a possible promoter of tumor metabolism and make those tumor cells with high expression of ENO1 have growth advantage [27]. SRD5A3 is considered to be a target of prostate cancer treatment [28]. However, we did not find the relationship between PAM, metabolism, and tumor. The traditional prognosis system usually does not estimate the risk stratification and clinical results accurately. Thus, the prediction method dependent on 8-mRNA markers will help predict metastasis and prognosis of HCC in contrast with one widely used biomarker.

Aerobic glycolysis is one of the most important characteristics of tumor, which provides survival advantage for tumor, also known as the “Warburg effect” [29]. This seemingly uneconomical way of energy supply is necessary for tumor cells. It not only provides energy for the growth of tumor cells but also provides raw materials for their biosynthesis. Zuo et al. believed that PGC1-*α* can inhibit the Warburg effect by downregulating pyruvate dehydrogenase kinase isoenzyme 1 (PDK1), so PGC1-*α* is considered as a potential factor to predict the prognosis and treatment target of liver cancer patients [4]. In terms of treatment, Ma et al. found that SRC-3 was highly expressed in patients with liver cancer and interacted with c-myc, the central regulator of the Warburg effect to promote its recruitment to the glycolysis gene promoter. And SRC-3 maybe is a potential target for sorafenib resistant treatment of liver cancer [30]. Three key allosteric enzymes control aerobic glycolysis: hexokinase (HK), phosphofructokinase (PFK), and pyruvate kinase (PK). Hexokinase (HK) is a key enzyme that catalyzes the first irreversible step of glycolysis and is associated with poor prognosis in cancer patients. Xu et al. found that mir-885-5p can act on 3' UTR of hexokinase 2 (HK2), significantly reducing glucose uptake and lactate production [31]. PFK is highly expressed and activated in human cancers, including HCC, in order to generate additional energy to support tumor growth. PFK is an important potential target that can take away from cancer cells the energy and matrix needed for macromolecular synthesis and proliferation and cause normal cells to survive. The last main enzyme in glycolysis is pyruvate kinase (PK). Among the three key enzymes, PK is the most important because it controls the final conversion of phosphoenolpyruvate to pyruvate. There are four subtypes of PK (*L*, *R*, *M*1, and *M*2), and PKM2 has been found to increase significantly in hepatoma cells and played a key role in the regulation of glycolysis. It has been reported that targeting PKM2 can enhance the therapeutic effect of HCC. In general, these important glycolytic enzymes play an important role in the growth and treatment of HCC. Glycolysis can therefore be involved in the development and occurrence of HCC. We reported first a glycolysis gene marker (PAM, NUP155, KDELR3, NSDHL, ENO1, SRD5A3, GOT2, and PKM) and then proved the prognostic value for HCC.

In conclusion, eight gene risk factors associated with glycolysis are reported which can help predict survival and prognosis in HCC patients. The greater the probability factor, the bad the prediction is. This finding will allow prospective studies to discover potential HCC treatments which will provide HCC patients with further genomic targets.

## Figures and Tables

**Table 1:** Clinical pathological parameters of patients with HCC in this research.

Clinical characteristic	*N*	%
Age (years)		
≤65	235	64.2
>65	131	35.8

Gender		
Male	255	67.6
Female	122	32.4

Grade		
I-II grade	235	63.2
III-IV grade	137	36.8

Stage		
I-II stage	262	74.2
III-IV stage	91	25.8

*T* classification		
T1-T2	280	74.9
T3-T4	94	25.1

*M* classification		
M0	272	98.6
M1	4	1.4

*N* classification		
N0	257	98.5
N1	4	1.5

**Table 2:** Gene sets enriched in hepatocellular carcinoma (377 samples).

HCC follow link to MSigDB	Size	ES	NOM *P* value	Rank at Max.
BIOCARTA_GLYCOLYSIS_PATHWAY	3	0.752	0.224	4380
HALLMARK_GLYCOLYSIS	199	0.525	*P* ≤ 0.001	11805
KEGG_GLYCOLYSIS_GLUCONEOGENESIS	62	−0.372	0.145	1079
REACTOME_GLYCOLYSIS	71	0.624	0.005	10406
REACTOME_REGULATION_OF_GLYCOLYSIS	12	0.621	0.037	8630

**Table 3:** The detailed information of eight independent prognostic mRNAs significantly associated with overall survival in patients with hepatocellular carcinoma.

mRNA	Ensemble ID	Location	Β (Cox)	HR
PAM	ENST00000438793	Chromosome 5: 102,201,430–102,366,809	0.219257548	1.245151921
NUP155	ENST00000231498	Chromosome 5: 37,288,239–37,371,283	0.454216998	1.574939719
GOT2	ENST00000245206	Chromosome 16: 58,741,035–58,768,261	−0.283535511	0.753116378
KDELR3	ENST00000216014	Chromosome 22: 38,864,067–38,879,452	0.139601816	1.14981587
PKM	ENST00000335181	Chromosome 15: 72,491,373–72,523,547	−0.178542466	0.836488534
NSDHL	ENST00000370274	Chromosome X: 151,999,511–152,038,273	0.320316797	1.377564104
ENO1	ENST00000234590	Chromosome 1: 8,921,061–8,938,749	0.182914904	1.200712228
SRD5A3	ENST00000264228	Chromosome 4: 56,212,276–56,239,263	0.313286399	1.367913244

**Table 4:** Univariable and multivariable analyses for each clinical feature.

Clinical feature	Univariate analysis			*P* value	Multivariate analysis			*P* value
	HR	HR.95L	HR.95H		HR	HR.95L	HR.95H	
Age	1.009	0.994	1.024	0.235	1.007	0.993	1.022	0.302
Gender	0.812	0.553	1.194	0.291	0.846	0.572	1.250	0.400
Grade	1.143	0.889	1.469	0.298	1.048	0.802	1.368	0.733
Stage	1.622	1.326	1.986	≤0.001	1.485	1.192	1.849	≤0.001
RiskScore	1.927	1.587	2.339	≤0.001	1.770	1.428	2.195	≤0.001

## Data Availability

The datasets used to support the findings of this study are available from TCGA (https://portal.gdc.cancer.gov/repository) database.

## References

[1] Torre L. A., Bray F., Siegel R. L., Ferlay J., Lortet-Tieulent J., Jemal A. (2015). Global cancer statistics, 2012. *CA: A Cancer Journal for Clinicians*.

[2] Du Y., Lu S., Ge J. (2020). ROCK2 disturbs MKP1 expression to promote invasion and metastasis in hepatocellular carcinoma. *American Journal of Cancer Research*.

[3] Guichard C., Amaddeo G., Imbeaud S. (2012). Integrated analysis of somatic mutations and focal copy-number changes identifies key genes and pathways in hepatocellular carcinoma. *Nature Genetics*.

[4] Zuo Q., He J., Zhang S. (2021). PPAR*γ* coactivator‐1*α* suppresses metastasis of hepatocellular carcinoma by inhibiting warburg effect by *PPARγ*-dependent WNT/*β*‐Catenin/Pyruvate dehydrogenase kinase isozyme 1 axis. *Hepatology*.

[5] Sayiner M., Golabi P., Younossi Z. M. (2019). Disease burden of hepatocellular carcinoma: a global perspective. *Digestive Diseases and Sciences*.

[6] Aravalli R. N., Steer C. J., Cressman E. N. K. (2008). Molecular mechanisms of hepatocellular carcinoma. *Hepatology*.

[7] Meyerson M., Gabriel S., Getz G. (2010). Advances in understanding cancer genomes through second-generation sequencing. *Nature Reviews Genetics*.

[8] Cho W., Ziogas D. E., Katsios C., Roukos D. H. (2012). Emerging personalized oncology: sequencing and systems strategies. *Future Oncology*.

[9] Singh A. K., Kumar R., Pandey A. K. (2018). Hepatocellular carcinoma: causes, mechanism of progression and biomarkers. *Current Chemical Genomics and Translational Medicine*.

[10] Zhang L., Zhang Z., Yu Z. (2019). Identification of a novel glycolysis-related gene signature for predicting metastasis and survival in patients with lung adenocarcinoma. *Journal of Translational Medicine*.

[11] Liu C., Li Y., Wei M., Zhao L., Yu Y., Li G. (2019). Identification of a novel glycolysis-related gene signature that can predict the survival of patients with lung adenocarcinoma. *Cell Cycle*.

[12] Wang Z.-H., Zhang Y.-Z., Wang Y.-S., Ma X.-X. (2019). Identification of novel cell glycolysis related gene signature predicting survival in patients with endometrial cancer. *Cancer Cell International*.

[13] Hanahan D., Weinberg R. A. (2011). Hallmarks of cancer: the next generation. *Cell*.

[14] Cheng G., Zielonka J., Dranka B. P. (2012). Mitochondria-targeted drugs synergize with 2-deoxyglucose to trigger breast cancer cell death. *Cancer Research*.

[15] Thomas M. A., Yang L., Carter B. J., Klaper R. D. (2011). Gene set enrichment analysis of microarray data from Pimephales promelas (Rafinesque), a non-mammalian model organism. *BMC Genomics*.

[16] Linehan W. M., Ricketts C. J. (2019). The Cancer Genome Atlas of renal cell carcinoma: findings and clinical implications. *Nature Reviews Urology*.

[17] Subramanian A., Tamayo P., Mootha V. K. (2005). Gene set enrichment analysis: a knowledge-based approach for interpreting genome-wide expression profiles. *Proceedings of the National Academy of Sciences*.

[18] IuS T. (1964). Detection of embryo-specific alpha-globulin in the blood serum of a patient with primary liver cancer. *Voprosy Meditsinskoi Khimii*.

[19] Pozzan C., Cardin R., Piciocchi M. (2014). Diagnostic and prognostic role of SCCA-IgM serum levels in hepatocellular carcinoma (HCC). *Journal of Gastroenterology and Hepatology*.

[20] Giannelli G., Fransvea E., Trerotoli P. (2007). Clinical validation of combined serological biomarkers for improved hepatocellular carcinoma diagnosis in 961 patients. *Clinica Chimica Acta*.

[21] Bao Z.-S., Li M.-Y., Wang J.-Y. (2014). Prognostic value of a nine-gene signature in glioma patients based on mRNA expression profiling. *CNS Neuroscience & Therapeutics*.

[22] Cheng W., Ren X., Cai J. (2015). A five-miRNA signature with prognostic and predictive value for MGMT promoter-methylated glioblastoma patients. *Oncotarget*.

[23] Peng P.-L., Zhou X.-Y., Yi G.-D., Chen P.-F., Wang F., Dong W.-G. (2018). Identification of a novel gene pairs signature in the prognosis of gastric cancer. *Cancer Medicine*.

[24] Holzer K., Ori A., Cooke A. (2019). Nucleoporin Nup155 is part of the p53 network in liver cancer. *Nature Communications*.

[25] Li Y., Yang X., Yang J., Wang H., Wei W. (2018). An 11‐gene‐based prognostic signature for uveal melanoma metastasis based on gene expression and DNA methylation profile. *Journal of Cellular Biochemistry*.

[26] Semenza G. L. (2010). Defining the role of hypoxia-inducible factor 1 in cancer biology and therapeutics. *Oncogene*.

[27] Cheng Z., Shao X., Xu M., Zhou C., Wang J. (2019). ENO1 acts as a prognostic biomarker candidate and promotes tumor growth and migration ability through the regulation of Rab1A in colorectal cancer. *Cancer Management and Research*.

[28] Uemura M., Tamura K., Chung S. (2008). Novel 5 alpha-steroid reductase (SRD5A3, type-3) is overexpressed in hormone-refractory prostate cancer. *Cancer Science*.

[29] Vander Heiden M. G., Cantley L. C., Thompson C. B. (2009). Understanding the Warburg effect: the metabolic requirements of cell proliferation. *Science*.

[30] Ma L., Liu W., Xu A. (2020). Activator of thyroid and retinoid receptor increases sorafenib resistance in hepatocellular carcinoma by facilitating the Warburg effect. *Cancer Science*.

[31] Xu F., Yan J.-J., Gan Y. (2019). miR-885-5p negatively regulates warburg effect by silencing hexokinase 2 in liver cancer. *Molecular Therapy-Nucleic Acids*.

